# The Opening of the Medical Schools

**Published:** 1910-09-24

**Authors:** 


### THE OPENING OF THE MEDICAL SCHOOLS.

The opening of the winter session of St. Mary's Hospital Medical School will take place on Monday, October 3, at 3 o'clock, when the introductory address will be given by Sir Arthur Conan Doyle, M.D., LL.D., who will also present the prizes and awards for the past session.

The opening of the seventy-sixth winter session of the Middlesex Hospital Medical School will take place on Monday, October 3, at 3 p.m. W. Salmon Nowell, Esq., M.A., M.R.C.S., L.D.S., will deliver the introductory address, after which the prizes gained during the previous year will be distributed by Lord Kitchener.

At Guy's Hospital the opening of the medical session will be, as usual, by the first meeting of the Physical Society. This year it will be held on Friday, October 7, and will take the form of a conversazione instead of dinner and address. Sir Samuel Wilks, Bart., the veteran Hon. President of the Society, will be present, if possible, to receive the guests, and will be supported by the Vice-Presidents, who are all on the consulting staff of the hospital. There will be the usual exhibits of interesting scientific instruments and of recent methods of investigation in the rooms above the Wills' library.

The opening of the next winter session at the various London hospitals will nearly everywhere take place on October 3. At Charing Cross Hospital Lady Juliet Duff will distribute the students' prizes, and Dr. F. W. Mott, physician to the hospital, will deliver the Eighth Biennial Huxley Lecture. Sir Thomas Barlow, President of the Royal College of Physicians, will preside at the opening ceremony at University College Hospital, and the Dean of Salisbury is to distribute the prizes. At the London School of Medicine for Women an address will be delivered by Mr. E. W. Roughton, surgeon to the Royal Free Hospital, on "Woman's Sphere in Medicine." At St. Bartholomew's Hospital an old students' dinner is to be held in the Great Hall of the hospital.

